# Culture-Specific Observations in a Saudi Arabian Digital Home Health Care Program: Focus Group Discussions With Patients and Their Caregivers

**DOI:** 10.2196/26002

**Published:** 2021-12-08

**Authors:** Abdulaziz A Alodhayani, Marwah Mazen Hassounah, Fatima R Qadri, Noura A Abouammoh, Zakiuddin Ahmed, Abdullah M Aldahmash

**Affiliations:** 1 Department of Family and Community Medicine College of Medicine King Saud University Riyadh Saudi Arabia; 2 Prince Naif Bin Abdulaziz Health Research Center King Saud University Riyadh Saudi Arabia; 3 Medical Researches Company iResearch Riyadh Saudi Arabia; 4 Riphah Institute of Healthcare Improvement & Safety Riphah International University Islamabad Pakistan; 5 Stem Cell Unit, Department of Anatomy College of Medicine King Saud University Riyadh Saudi Arabia; 6 College of Medicine Vision Colleges Riyadh Saudi Arabia

**Keywords:** connected health, digital health, telehealth, telemedicine, culture, Islam, Arab, mobile phone

## Abstract

**Background:**

There is growing evidence of the need to consider cultural factors in the design and implementation of digital health interventions. However, there is still inadequate knowledge pertaining to the aspects of the Saudi Arabian culture that need to be considered in the design and implementation of digital health programs, especially in the context of home health care services for patients who are chronically and terminally ill.

**Objective:**

This study aims to explore the specific cultural factors related to patients and their caregivers from the perspective of physicians, nurses, and trainers that have influenced the pilot implementation of Remotely Accessible Healthcare At Home, a connected health program in the Home Health Care department at King Saud University Medical City, Riyadh, Saudi Arabia.

**Methods:**

A qualitative study design was adopted to conduct a focus group discussion in July 2019 using a semistructured interview guide with 3 female and 4 male participants working as nurses, family physicians, and information technologists. Qualitative data obtained were analyzed using a thematic framework analysis.

**Results:**

A total of 2 categories emerged from the focus group discussion that influenced the experiences of digital health program intervention: first, culture-related factors including language and communication, cultural views on using cameras during consultation, nonadherence to web-based consultations, and family role and commitment and second, caregiver characteristics in telemedicine that includes their skills and education and electronic literacy. Participants of this study revealed that indirect contact with patients and their family members may work as a barrier to proper communication through the Remotely Accessible Healthcare At Home program.

**Conclusions:**

We recommend exploring the use of interpreters in digital health, creating awareness among the local population regarding privacy in digital health, and actively involving direct family members with the health care providers.

## Introduction

### Background

Digital health technology (including telemedicine, telehealth, remote patient monitoring, and connected health) that supports patient-centered care is a rapidly evolving field and is gaining popularity as a means of driving quality improvements and accessibility in health care [1]. Digital health is assumed to enhance the provision of health care services outside conventional hospital settings [2,3], thereby strengthening the development of home health care (HHC) services. Numerous benefits related to the implementation of digital health have been suggested, including improved quality of care, patient safety, accessibility, and cost minimization [4,5]. Their use is also linked with enhanced clinical outcomes, such as a decline in mortality and emergency room admission rates [6]. During the 2019 COVID-19 global pandemic, telemedicine and telehealth have provided opportunities for delivering health care remotely and have underpinned the beneficial role of digital health in the future [7].

Owing to these beneficial uses of technology in health care worldwide, there has been a dramatic growth in the number of digital health pilot programs [8]. However, a critical issue in this domain is that a considerable number of these pilot programs have been unsuccessful in progressing toward full-scale implementation and widespread adoption [9-11]. The reasons underlying the failure of these pilot programs have received substantial interest in the literature [12,13]. One of the key barriers identified in numerous studies pertains to cultural factors and influences [14-16]. Culture refers to “a system of knowledge, beliefs, patterns of behavior, artifacts, and institutions that are created, learned, and shared by a group of people” [17]. Culturally appropriate health-related interventions have been reported to improve clinical outcomes [18]. Likewise, cultural sensitivity is a key element for honoring patient-centered health care [19], and it has been identified as one of the significant factors needed to extend telemedicine projects to mainstream health care [20].

Being the cradle of the Islamic faith, Saudi Arabia is home to a predominantly Muslim population, and this shapes cultural and social life. In general, some characteristics of the Arab-Muslim culture that influence health include health beliefs, family relationships, health providers, diet and medications, life stages, views on death and dying, preventive health, gender issues, space, time, and communication [21]. Specific to Saudi Arabia, the language barrier between patients and providers has been well documented to adversely affect the quality of health care in Saudi Arabia [22,23].

The influence of culture on digital health practices in Saudi Arabia is the focus of a study by Kaliyadan et al [24], which included 166 patients and evaluated the application of a 4G smartphone for teledermatology. The study found that 14% of the mostly female patients refused photography of their skin lesions, citing social and cultural reasons [24]. In addition, a review article by Alkhalifah and Aldhalaan [25] that examined the use of telehealth services to support families with children diagnosed with autism spectrum disorder in rural areas of Saudi Arabia recognized that telehealth interventions developed in the West were culturally unsuitable in the local context, thus acting as a barrier to extensive adoption [25]. Hence, the provision of culturally appropriate digital health is crucial for delivering high-quality health care in Saudi Arabia.

### Research Gap

There is still inadequate knowledge pertaining to the aspects of Saudi Arabian culture that need to be considered closely in the design and implementation of digital and connected health programs. This is especially relevant in the context of HHC services for vulnerable groups such as patients who are chronically and terminally ill that require longstanding treatment and management and often have complex unmet health care needs [26]. Information regarding the unique cultural factors of the patients and caregivers that influence the successful application and adoption of health technology will enable program managers and health care providers to design and deliver culturally sensitive digital health interventions for use in the local context [27]. This is especially vital in light of Saudi Arabia’s Vision 2030 National Transformation Program (2018-2020) that has reinforced the role of patient-centered telemedicine and digital health technology as a means of driving major developments in the health care sector and improving health care outreach [28]. Its vitality is also relevant in light of Saudi Arabia’s active digital health response to the COVID-19 pandemic [29].

Thus, through our study, we aim to explore the culture-specific observations relating to patients and their caregivers through the perspective of physicians, nurses, and trainers during the pilot implementation of a connected health program named Remotely Accessible Healthcare At Home (RAHAH) in the HHC department at King Saud University Medical City (KSUMC), Riyadh, Saudi Arabia.

## Methods

### Study Design

This is a phenomenological qualitative study using focus group discussions (FGDs) to explore experiences and opinions of health care workers (HCWs) and program trainers who have been using telehealth for HHC in the Saudi Arabian cultural context. FGD was used as the technique, and it reflects the process through which meaning was constructed collectively and can be regarded as naturalistic.

Some of the researchers (AAA, AMA, ZA, and FRQ) held an emic perspective as they worked in close contact with the RAHAH team. This assisted in bringing more knowledge, as they were more familiar with the expressions and ways of communicating and establishing trust between the researchers and participants. The others held an etic perspective (MMH and NAA), as they were only involved for the purpose of conducting the study. This allowed them to look at the participants experiences without a preconceived assumption.

### Study Setting

**Figure 1:**
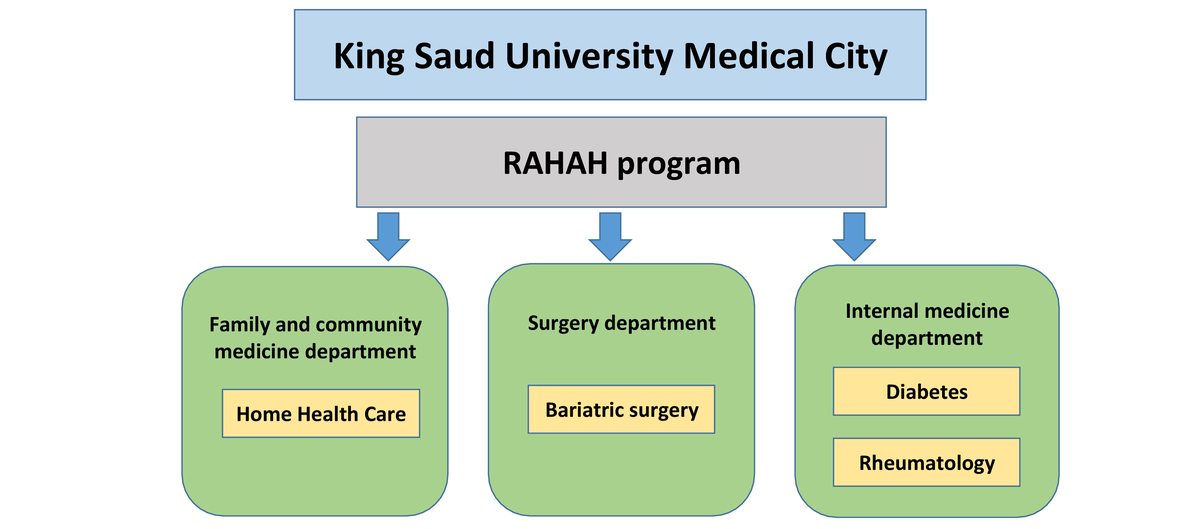
Different departments piloted by Remotely Accessible Healthcare At Home program including the Home Health Care department. RAHAH: Remotely Accessible Healthcare At Home.

KSUMC is a tertiary care hospital in the capital city of Riyadh. It is a leader in medical specialties, subspecialties, and technologies. A connected health program called *RAHAH* was developed by Prince Naif Bin AbdulAziz Health Research Center at KSUMC. RAHAH uses a wide array of digital-based health tools on its platform, including patient health records, portals, mobile apps, remote monitoring devices (such as glucometers, blood pressure monitors, oximeters, and thermometers), teleconsultation, and e-response centers (through messaging and chat). In 2018, the HHC department at KSUMC embarked on piloting the RAHAH program in its department (Figure 1). This pilot program is being conducted in a phased approach, with geriatric patients constituting the first phase. There were 40 geriatric patients with chronic conditions such as diabetes mellitus and hypertension who were enrolled in this program when the study was conducted.

### Sampling and Recruitment

This research project was conducted at the HHC department, KSUMC where 6 HCWs were dedicated to piloting the RAHAH program. RAHAH provides technical liaisons to different departments at KSUMC to implement the RAHAH program. These liaisons also train patients, caregivers, physicians, nurses, and allied health staff on how to use the service, observe and curate feedback from users, and help in cases of technical hurdles. They provide valuable insight into the audience of interest (the HHC team and patients or caregivers) because of their close and frequent contact with them. For the HHC department at the time of this study, there were 2 RAHAH technical liaisons. This project’s research team comprises administrative researcher physician figures from the HHC department and RAHAH program (AAA, AMA, and ZA), 2 qualitative research public health physicians from community medicine units (MMH and NAA), and a RAHAH researcher (FRQ).

The criteria for participant selection included those working on the RAHAH project in the HHC department at KSUMC. Those who were not directly involved in the HHC department at KSUMC were excluded. The total number of team members working on the RAHAH pilot in the HHC department was 8 (6/8, 75% HCWs and 2/8, 25% technical liaisons). They were all invited by researchers MMH and FRQ (who are not associated with HHC) via email to participate in the focus group. It was explained to all participants that their decision to participate would not have any implication on their jobs. An invitee declined (1 of the 2 RAHAH technical liaisons), and the remainder accepted by responding to the email. Thus, a total of 7 participants attended the FGD. Reminders for the invitation date and time were sent out via SMS text messages and emails with no prior knowledge of the actual number that will be attending the focus group. The focus group time was voided from the HHC team schedule to provide an opportunity to those who wanted to participate in the research.

### Data Collection

The discussion guide was developed in English based on the study objectives, previous studies [15,21,30-33], professionals’ experiences [34], and personal familiarity and clinical experiences with the Saudi Arabian culture (Multimedia Appendix 1). It followed a semistructured format with open-ended questions that facilitated the development of emergent themes during the discussion.

The discussion guide was tested with 1 RAHAH trainer who was not invited to the FGD. A researcher tested the guide for ease of understanding and checked whether the answers met the requirements of the question. Consequently, some of the guide questions were rephrased, and the definition of *culture* was added to the introduction.

The FGD was conducted on July 28, 2019, for 2 hours by 2 researchers, MMH and FRQ. MMH led the discussion, as she is bilingual in Arabic and English, whereas FRQ took notes of the discussion and kept track of the time. The discussion was conducted in a quiet meeting room at KSUMC on July 28, 2019, and lasted for an hour.

The discussion was in English, which is the main language spoken by workers in KSUMC. However, to enhance the credibility of the findings, participants were given the option to use Arabic. For each question, the focus group facilitator gave ample time for all participants to share their thoughts and at times used prompters to further explore the answers. The facilitator would move on to the next question when she reached saturation with the answers.

The discussion was audiotaped and transcribed verbatim by a transcription service. The transcript had time stamps on speaker change, and MMH and FRQ filled in unintelligible and Arabic audio, deidentified the participants and names, crosschecked the text with audio, and refined and finalized the transcript for analysis.

Having different perspectives among the authors, using probing questions, using ≥1 coder, and having transparency in reporting the results added to the trustworthiness of the study. A focus group method was chosen as it is the best technique to elicit shared experiences, permitting participants to raise topics that they deem to be important and allowing people to probe each other’s reasons for holding certain views. All of the above enriched the data and supported the credibility of the study.

### Data Analysis

We conducted a thematic framework analysis as described by Ritchie and Spencer [35]. A holistic overview of the data set and familiarization with the range, depth, and diversity of the participants’ responses was performed by NAA and MMH. Similar codes were developed by both authors, and an agreement on an initial coding frame was achieved with the flexibility to enable other codes to be added and discussed regularly. Key themes were developed, and quotations were used to support the provided evidence. NVivo software (version 11.4.2; QSR International) [36] was used to manage the data.

### Ethical Considerations

Ethical approval was obtained from the institutional review board of King Saud University College of Medicine in June 2019 (project number E-18-3914) based on the Declaration of Helsinki. Participation in the study was voluntary, maintaining the right of the participants to withdraw at any time. The participants were fully informed regarding the study aims, objectives, methods, and how the interview material would be recorded and protected. All the information provided by the participants was kept confidential, the participants’ names were coded, and possible identifiers were omitted from the final transcript. As the head of the HHC department and RAHAH program managers are researchers in this study, they were not involved in the facilitation or transcription of this focus group to avoid the power effect of the relationship between managers and employees to influence the latter’s responses.

## Results

### Overview

A total of 7 participants were included in the focus group (Table 1), and 2 categories emerged from the FGD. These were (1) culture-related factors, including language and communication, cultural views on using cameras during consultation, nonadherence to web-based consultations, and family role and commitment and (2) caregiver characteristics in telemedicine, including their skills, education, and electronic literacy (Textbox 1).

**Table 1:** Demographic data of the study participants.

Participant number	Nationality	Gender	Time working in home care and telemedicine	Specialty
1	Saudi Arabia	Female	3 years	Information technology
2	South Asia	Male	3 years	Family physician
3	South Asia	Male	1 year	Family physician
4	Southeast Asia	Male	4 years	Nurse
5	Southeast Asia	Male	3 years	Nurse
6	South Asia	Female	3 years	Nurse
7	Saudi Arabia	Female	3 months	Nurse

**Emerging themes in the experiences of health care workers.**

**Culture-related factors**

- Language and communication
- Communicating with the caregiver
- Behavior and media used in urgent communication
- Bilingualism
- Cultural views on using the camera
- Nonadherence to web-based consultations
- Family role and commitment

**Caregiver characteristics**

- Skills and education
- Electronic literacy

### Culture-Related Factors

A total of 4 themes emerged from this category: language and communication, cultural views on using the camera, nonadherence to web-based consultations, and family role and commitment.

#### Language and Communication

#### Communicating With the Caregiver

The participants acknowledged that dealing with the caregivers rather than directly with patients is inevitable, as almost all patients who are registered in the RAHAH program at the HHC department are diagnosed with cognitive impairment. This is evident in the response of participant 2:

Most of the patients are not able to express themselves...they are demented, and they cannot express themselves.

As family members are usually busy during the day, they assign a housemaid or sometimes a nurse to escort the patient. A participant explained the following: “We are communicating with the attendants who are hired ones, not the real relatives of the patient” [participant 2].

Another participant added the following: “Very few, uh, family members are communicating with us” [participant 1].

Caregivers were seen by the participants as an essential link between the patients and the health care team. According to them, they train all caregivers to assist in patient care: “We train them on feeding, giving medications, vital signs recording” [participant 2].

They correspondingly acknowledged that if the caregiver was sincere, good patient outcomes could be observed. A participant explained as follows:

Dedication is very important for the caregiver because we have some patients with bed sores...So, if she is dedicated [you can see] good progress and good healing of the wound...But if they are not dedicated and they are not concerned, it will linger on.

participant 3

Furthermore, discrepancies in the information shared by patients and caregivers were noted. A participant explained the following:

Even when their caregivers told us that she [the patient] was having pain in her knees, she was not eating, she—didn’t go to the toilet for like three, four days and I asked her, “Mama, how are you? Are you okay?” “Yeah, everything is good.”

participant 7

Moreover, handover of care between caregivers and family members was described as *poor,* as they do not involve the health care team in the process of care transition to family members. A participant stated the following: “They [caregivers] will not even tell us when they are travelling” [participant 7].

#### Behavior and Media Used in Urgent Communication

According to the participants, phone calls to their offices or messages on WhatsApp were the modes of communication in case of emergencies. A participant explained the following:

We are not watching RAHAH all the time...They [patients] call our office. If there is some urgent problem...they send the pictures, messages on WhatsApp and immediately we connect and then call them and see what the problem is.

participant 2

Participants felt comfortable providing their personal cell phone contacts, as they believed that some patients might need immediate attention:

If the nasogastric tube is out...So, they communicate with us because our patients need to be fed...or if the catheter is pulled out, they need to be relieved.

participant 3

A participant expressed his inconvenience in dealing remotely with some caregivers in the case of an emergency. He believed that caregivers’ expressed stress could be exaggerated, and the situation could be misinterpreted by the HCW:

You directly see they are very hysterical, especially ladies, and shouting “the patient is dying! The patient is dying!” or “there’s blood coming out” or “please, we cannot give medicine anymore.” So, once they are hysterical...They do create a hysterical situation for the treatment team.

participant 2

#### Bilingualism

The participants denied language as a barrier to communication. They noticed that their caregivers or relatives spoke both Arabic and English, which made it easier for them to communicate. Participant 3 explained it as follows: “If you go back years and years, most people were speaking Arabic, but now I have noticed most of the people there speak English fluently too.*”*

In contrast, another participant noted the following:

Sometimes they do not know how to use the system because the RAHAH system is an English based system...Others [caregivers] from African countries do not speak English, so they do not communicate with us directly and they just work under the supervision of some ladies of the house.

participant 2

The problem of caregivers with limited knowledge of the English language contributes to difficulties in communication with health care providers and eventually influences the level of care provided to the patient. Those who are fluent in English would be able to operate the telehealth program effectively and understand the treatment team instructions better compared with those who do not speak English:

Some female foreigner caregivers can speak English and they can operate the telehealth system. They are the most helpful to us, taking pictures, and sending us messages.

participant 2

Others are housemaids who are not trained in the English language and do not communicate well with us; they just work under the supervision of family members. They are trained to clean the patient’s bed, bedsheets, and things, and they are not trained to take care of medical problems. They are also not able to use the telemedicine system.

participant 2

#### Cultural Views on Using the Camera

Participants commented on the acceptance of camera use by patients during web-based consultations. A participant noted the following:

Even if I ask them to show me the patient, they are reluctant. They say “I will tell you everything instead.”

participant 2

Some participants believed that this is rooted in a religious viewpoint, acknowledging that Saudi Arabian culture is shaped by the Islamic religious background. A participant remarked the following: “Some might think that showing themselves on camera is Haram [religiously prohibited]” [participant 1].

Participants also speculated that most female patients were uncomfortable showing themselves on camera, as they covered their faces from male strangers. A participant further opined that the patients or caregivers feared using the camera and technology during communication with their health care provider and expressed concerns regarding their privacy:

They do not want to use the camera...because it could be recorded. They are afraid that technology is so advanced, and cameras can record their (face)...stalking...taking photos.

participant 2

The participants showed full acceptance of the patients’ preferences. Moreover, most of them preferred not to ask female patients to show themselves on camera anymore, as they knew this might not be acceptable to them and only tried to see parts of the body when necessary. Male patients, on the other hand, do not generally have reservations in this regard. A participant noted the following:

We do not request any exposure on camera. For females, we do not request to examine them on the telemedicine system...or to show parts of their body unless absolutely necessary. But in males...it is okay.

participant 3

#### Nonadherence to Web-Based Consultations

Participants perceived that patients’ attitudes toward attending their web-based appointments were different from face-to-face hospital appointments. Some patients do not take their web-based appointments seriously. A participant explained the following:

They are not particular about this appointment and whether to attend it or not...In out-patient clinics, people are careful because if they do not show up then they will not get another appointment for three months. Here, there is no such problem. They can get another one soon.

participant 2

#### Family Role and Commitment

Most participants acknowledged the key role of family members in facilitating the implementation of the RAHAH program in HHC and communicating with health care providers. Moreover, their role in reaching an optimum state of health care outcomes was highlighted. The participants mentioned the following:

The main role is the family’s.

participant 3

If the family is proactive, we can expect that the patient has no acute conditions...their health status is optimum and meet expectations.

participant 5

However, the participants conveyed that family members were unavailable, most of the time, to take care of the patients:

...because family members are working outside the home, and they are busy.

participant 3

Men generally are not at home...Even ladies are working, such as teachers.

participant 2

Consequently, physicians generally rely on a hired nonnational caregiver to provide them with health-related information about the patient, and sometimes, the team may find it difficult to communicate with family members when the caregiver is unavailable. A participant noted the following:

They [the family member] will be updated by the caregiver, usually a housemaid. And whenever we ask more questions, they need to call the caregiver for more clarification. This may create a problem with telling the truth.

participant 7

The participants further acknowledged that the commitment of families varied regarding their involvement in patient care. A participant reported a few incidents that highlighted the lack of commitment by some family members:

Do you remember what happened last week with us? The patient who had not taken medicine...he used to take medicine at 9 or 10 AM. Why? Because the caregiver was traveling. The lady of the house was there at home, and she said, “I don’t know how to give the medications. I told the caregiver that before she leaves, she should hand over the care.”

participant 3

A participant reported that some families hindered the successful implementation of the telehealth program and recounted an instance in which the family did not provide the Wi-Fi password to the caregiver in their absence:

I have encountered two caregivers who want to use RAHAH, but the problem is they do not have Wi-Fi. I mean the family do not give the password to the caregivers.

participant 4

In short, the quality of the telehealth experience is affected by specific culture-related factors, including language and mode of communication, religious views of the patients or caregivers, and family role and commitment to patients’ care.

### Caregiver Characteristics

As caregivers are the main link between patients and health care providers, under this category, caregiver characteristics that were commonly observed by the participants were classified into the following themes: skills and education and electronic literacy of the caregiver.

#### Skills and Education

The participants highlighted several individual skills of the caregivers that influenced the care provided to patients. For example, a participant noted the following:

We see all types and all categories of caregivers. Some are trained nurses, some are nursing assistants, and some are only housemaids.

participant 3

Educated caregivers prove to be valuable in driving an effective telehealth consultation, as they are able to identify dangerous signs and symptoms in the patient and inform the treating team in a timely manner. A participant described incidents where an educated caregiver was able to use the telehealth program to connect with the health care team and alert them regarding unusual symptoms such as blood in the stool and discoloration of the patient’s leg, which subsequently saved the life of the patient:

So, an educated attendant is the main person who is helping us in telemedicine...remember that lady? She sent out the photos of the blood in the stool. Therefore, we immediately contacted her and then we went there to see the patient, and found out that he had colon cancer...There was a blood clot in the leg that we recognized from picture that she sent.

participant 2

#### Electronic Literacy

According to the participants, some caregivers had limited electronic literacy and found it difficult to deal with the technology necessary to conduct a telehealth consultation:

Yeah, um, few times I faced difficulties in leading the caregiver to just sign up to Gmail...even the application...sometimes they have difficulties with that.

participant 1

In addition, some patients and caregivers showed low self-confidence in using the technology. A participant explained the following:

One patient said “I do not know how to use the application. I do not know how to use technology. I am not good with phones.”

participant 7

## Discussion

### Principal Findings

**Figure 2:**
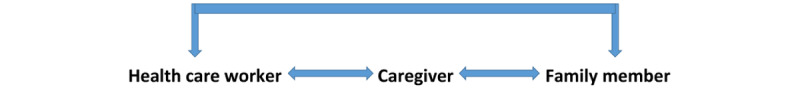
Pathway of health communication using telemedicine in Remotely Accessible Healthcare At Home program in the Home Health Care department.

The RAHAH program applies a user-centered design approach to its development process. The program team is in constant communication with the health care providers piloting it, as well as patients and caregivers receiving care through the program. The program team also conducts frequent surveys and observation visits to better understand the user experience and enhance it. This qualitative study aligns with this approach of adopting the bottom-up design of the program for better acceptability, usability, and satisfaction. The pathway of communication in the RAHAH program is depicted in Figure 2, where the HCWs communicate with the caregiver, as most patients have difficulties in verbal communication, who in turn communicate with the family members. HCWs communicate with family members only when needed or in emergency cases.

Our findings show that HCWs communicate directly with caregivers who are characterized by some features that may hinder proper telemedicine-based communication and hence affect the quality of care provided by the health care team. This finding aligns well with previous studies that suggested cultural influences impede the full-fledged implementation and adoption of digital health systems [10-12]. Moreover, although HCWs using telemedicine believe that better health outcomes are associated with family involvement, their direct communication with the family members of the patients is lacking. This can be explained by the changing social culture in Saudi Arabia in terms of more women joining the workforce [37] and the breakdown of the extended family structure [38].

Although language was not explicitly reflected as a significant barrier by the participants, this aspect must be considered because the probability of challenges related to language and communication is high in Saudi Arabia, as many of the caregivers are expatriates and do not speak Arabic [22,23]. This is a matter of concern, as poor patient–provider communication is linked to poor diagnosis, treatment, and medication instructions and a significantly higher risk of serious medical events [39-43]. Bilingual providers are preferred to bridge the patient–provider gap. In this regard, researchers suggest that professionally trained interpreters can provide a high-quality, culturally competent language in the absence of the services of bilingual clinicians [44]. However, a scarcity of interpreters has been reported previously [45], and in Saudi Arabia, a systematic review suggested that the cultural and language training currently provided to expatriate nurses is not fulfilling its purpose [46].

The findings of this study are consistent with earlier studies that highlight the cultural beliefs affecting camera use in teleconsultations, especially among women [24,47-49]. The culture-religious belief that inhibits women from showing themselves on camera is deeply rooted in decades-old *Fatwas* (scholarly religious decisions) that forbade photography in all its forms and the revealing of any part of a female’s body to men, even in medical situations. These *Fatwas* have been amended to accommodate the modern-day context but remain a deterrent in the consciousness of some patients. It was clearly important for the participants of this study to be culturally sensitive when delivering their services using RAHAH. However, they may hinder a successful telehealth experience by refraining from asking female patients to appear on camera or to show some body parts.

Studies have largely reported technology-learning barriers in telemedicine [50-52]. Our findings contrast with previously published studies in which a positive attitude toward telemedicine was evident in patients with cancer [53], as well as in those with lung diseases [54]. In their large survey study, Edwards et al [55] found that patients with depression and those with a high risk of cerebrovascular disease have a moderate interest in phone-, email-, and internet-based services, whereas interest in social media–based services was lower. In Saudi Arabia, it was found that several barriers hinder decision-makers of health care facilities from adopting and implementing telemedicine in their health care facilities. These barriers were the availability of adequate, sustainable funding and financial support for operations; the reimbursement for telemedicine services; the quality of information; the cost-effectiveness of telemedicine; and cultural and social constraints [56]. Another local study highlighted factors such as privacy, equipment cost, lack of training, information, and communication technology issues as significant barriers to the adoption of telemedicine in hospitals [57]. However, the latter studies focused on patients or HCWs mediating the consultation in the absence of caregivers.

Regarding the recurring *no show* behavior, it could be curbed with punitive procedures and communicated to caregivers when onboarding the program. The cost-effectiveness of the implemented telehealth program should be assessed with regard to nonadherence to web-based consultations [58]. The *no show* behavior in the served population in this study could be explained by proximity to the hospital premises. In this case, telehealth was not the only option for patients. Telehealth services for individuals living in rural areas have observed different behavior [25,59].

Overall, it is important to empower patients and caregivers in the context of digital health care. Our study can be looked at as a piece of a whole. The whole picture includes patients, caregivers, and HCWs. The findings of this study can be compared against perspectives on the experience of the primary audience of the RAHAH program later on to improve the overall communication process.

### Study Limitations

Potential information recall bias among participants must be considered in similar studies in the future. This study would have benefited from triangulation with the primary audience (patients and caregivers). In this study, patients were not interviewed because of their health conditions, whereas caregivers spoke different languages, which required unavailable resources for translation to conduct the interviews and transcribe them. Future projects could triangulate with other departments’ findings and RAHAH’s user analytics and should capture the experiences of patients or their family members. The study team dedicated effort to concealing the participants’ identities by not involving research team members who are also HHC or RAHAH administrative figures in the focus group or analysis phases.

### Conclusions

The study results illustrate the importance of improving awareness among the public and health care teams to reassure the patients and caregivers that their privacy and preferences with respect to the socioreligious culture are vital. HCWs should remind caregivers of the importance of following a proper handover of care, should the need arise. Furthermore, because caregivers are not always professionally trained in nursing and personal characteristics affect their capabilities, it is crucial that HCWs communicate directly with family members, providing a summary of the teleconsultation conducted in their absence. More research could focus on the feasibility of using interpreters to improve the care provided via telehealth.

## Appendix

Multimedia Appendix 1 Focus group discussion guide.

## Abbreviations

FGD: focus group discussion
HCW: health care worker
HHC: home health care
KSUMC: King Saud University Medical City
RAHAH: Remotely Accessible Healthcare At Home

## References

[1] Chu L, Shah AG, Rouholiman D, Riggare S, Gamble JG, Rivas H, Wac K (2018). Patient-centric strategies in digital health. Digital Health. Health Informatics.

[2] Voran D (2015). Telemedicine and beyond. Mo Med.

[3] Vegesna A, Tran M, Angelaccio M, Arcona S (2017). Remote patient monitoring via non-invasive digital technologies: a systematic review. Telemed J E Health.

[4] Agboola S, Kvedar J (2016). Telemedicine and patient safety. AHRQ Patient Safety Network.

[5] Di Cerbo A, Morales-Medina JC, Palmieri B, Iannitti T (2015). Narrative review of telemedicine consultation in medical practice. Patient Prefer Adherence.

[6] Steventon A, Bardsley M, Billings J, Dixon J, Doll H, Hirani S, Cartwright M, Rixon L, Knapp M, Henderson C, Rogers A, Fitzpatrick R, Hendy J, Newman S, Whole System Demonstrator Evaluation Team (2012). Effect of telehealth on use of secondary care and mortality: findings from the Whole System Demonstrator cluster randomised trial. BMJ.

[7] Wosik J, Fudim M, Cameron B, Gellad ZF, Cho A, Phinney D, Curtis S, Roman M, Poon EG, Ferranti J, Katz JN, Tcheng J (2020). Telehealth transformation: COVID-19 and the rise of virtual care. J Am Med Inform Assoc.

[8] Colaci D, Chaudhri S, Vasan A (2016). mHealth interventions in low-income countries to address maternal health: a systematic review. Ann Glob Health.

[9] Huang F, Blaschke S, Lucas H (2017). Beyond pilotitis: taking digital health interventions to the national level in China and Uganda. Global Health.

[10] McCann D (2012). A Ugandan mHealth moratorium is a good thing. ICTworks.

[11] (2012). National eHealth Strategy Toolkit. International Telecommunication Union.

[12] Luna D, Almerares A, Mayan 3rd JC, González Bernaldo de Quirós F, Otero C (2014). Health informatics in developing countries: going beyond pilot practices to sustainable implementations: a review of the current challenges. Healthc Inform Res.

[13] Zanaboni P, Wootton R (2012). Adoption of telemedicine: from pilot stage to routine delivery. BMC Med Inform Decis Mak.

[14] Ly BA, Labonté R, Bourgeault IL, Niang MN (2017). The individual and contextual determinants of the use of telemedicine: a descriptive study of the perceptions of Senegal's physicians and telemedicine projects managers. PLoS One.

[15] Sundin P, Callan J, Mehta K (2016). Why do entrepreneurial mHealth ventures in the developing world fail to scale?. J Med Eng Technol.

[16] Chung J, Thompson HJ, Joe J, Hall A, Demiris G (2017). Examining Korean and Korean American older adults' perceived acceptability of home-based monitoring technologies in the context of culture. Inform Health Soc Care.

[17] Guest K (2014). Cultural Anthropology: A Toolkit for a Global Age.

[18] Zeh P, Sandhu HK, Cannaby AM, Sturt JA (2012). The impact of culturally competent diabetes care interventions for improving diabetes-related outcomes in ethnic minority groups: a systematic review. Diabet Med.

[19] Tucker CM, Marsiske M, Rice KG, Nielson JJ, Herman K (2011). Patient-centered culturally sensitive health care: model testing and refinement. Health Psychol.

[20] (2014). Eighteen critical success factors for deploying telemedicine. Momentum.

[21] Haddad LG, Hoeman SP (2000). Home healthcare and the Arab-American client. Home Healthc Nurse.

[22] Abouammoh NA, Barnes S, Goyder E (2016). Providing lifestyle advice to people with type 2 diabetes from different cultures: a qualitative investigation. Prim Care Diabetes.

[23] Alshammari M, Duff J, Guilhermino M (2019). Barriers to nurse-patient communication in Saudi Arabia: an integrative review. BMC Nurs.

[24] Kaliyadan F, Amin TT, Kuruvilla J, Ali WH (2013). Mobile teledermatology–patient satisfaction, diagnostic and management concordance, and factors affecting patient refusal to participate in Saudi Arabia. J Telemed Telecare.

[25] Alkhalifah S, Aldhalaan H (2018). Telehealth services for children with autism spectrum disorders in rural areas of the Kingdom of Saudi Arabia: overview and recommendations. JMIR Pediatr Parent.

[26] Herr M, Arvieu J, Aegerter P, Robine J, Ankri J (2014). Unmet health care needs of older people: prevalence and predictors in a French cross-sectional survey. Eur J Public Health.

[27] Aldahmash AM, Ahmed Z, Qadri FR, Thapa S, AlMuammar AM (2019). Implementing a connected health intervention for remote patient monitoring in Saudi Arabia and Pakistan: explaining 'the what' and 'the how'. Global Health.

[28] National Transformation Program Delivery Plan 2018-2020. Vision 2030.

[29] Hassounah M, Raheel H, Alhefzi M (2020). Digital response during the COVID-19 pandemic in Saudi Arabia. J Med Internet Res.

[30] Erwin DO, Treviño M, Saad-Harfouche FG, Rodriguez EM, Gage E, Jandorf L (2010). Contextualizing diversity and culture within cancer control interventions for Latinas: changing interventions, not cultures. Soc Sci Med.

[31] Mendieta M, Buckingham R, Kietzman J, Helal A, Parker S (2015). Understanding the barriers to hospice care in Saudi Arabia. J Palliat Med.

[32] Hilty D, Evangelatos G, Valasquez G, Le C, Sosa J (2018). Telehealth for rural diverse populations: cultural and telebehavioral competencies and practical approaches for clinical services. J Technol Behav Sci.

[33] Shore JH, Savin DM, Novins D, Manson SM (2006). Cultural aspects of telepsychiatry. J Telemed Telecare.

[34] Alotaibi HK (2019). Cultural considerations in patient experience. Proceedings of the 2nd International Patient Experience Summit.

[35] Wordpress.

[36] Unlock insights in your data with powerful analysis. NVIVO.

[37] (2020). Saudi women rising up in business in line with Vision 2030. World Bank Group.

[38] Almalki S, Ganong L, Robila M, Taylor AC (2018). Family life education in Saudi Arabia. Perspectives on Family Life Education.

[39] Al Shamsi H, Almutairi A, Al Mashrafi S, Al Kalbani T (2020). Implications of language barriers for healthcare: a systematic review. Oman Med J.

[40] Bonacruz Kazzi G, Cooper C (2003). Barriers to the use of interpreters in emergency room paediatric consultations. J Paediatr Child Health.

[41] Lasater LM, Davidson AJ, Steiner JF, Mehler PS (2001). Glycemic control in English- vs Spanish-speaking Hispanic patients with type 2 diabetes mellitus. Arch Intern Med.

[42] Shapiro J, Saltzer E (1981). Cross-cultural aspects of physician-patient communications patterns. Urban Health.

[43] Cohen AL, Rivara F, Marcuse EK, McPhillips H, Davis R (2005). Are language barriers associated with serious medical events in hospitalized pediatric patients?. Pediatrics.

[44] Juckett G, Unger K (2014). Appropriate use of medical interpreters. Am Fam Physician.

[45] Jaeger FN, Pellaud N, Laville B, Klauser P (2019). Barriers to and solutions for addressing insufficient professional interpreter use in primary healthcare. BMC Health Serv Res.

[46] Alshammari M, Duff J, Guilhermino M (2019). Barriers to nurse-patient communication in Saudi Arabia: an integrative review. BMC Nurs.

[47] Kuziemsky CE, Gogia SB, Househ M, Petersen C, Basu A (2018). Balancing health information exchange and privacy governance from a patient-centred connected health and telehealth perspective. Yearb Med Inform.

[48] Alshammari F, Hassan S (2019). Perceptions, preferences and experiences of telemedicine among users of information and communication technology in Saudi Arabia. J Health Inform Dev Ctries.

[49] Padela AI, Rodriguez del Pozo P (2011). Muslim patients and cross-gender interactions in medicine: an Islamic bioethical perspective. J Med Ethics.

[50] Sanders C, Rogers A, Bowen R, Bower P, Hirani S, Cartwright M, Fitzpatrick R, Knapp M, Barlow J, Hendy J, Chrysanthaki T, Bardsley M, Newman SP (2012). Exploring barriers to participation and adoption of telehealth and telecare within the Whole System Demonstrator trial: a qualitative study. BMC Health Serv Res.

[51] El-Mahalli AA, El-Khafif SH, Al-Qahtani MF (2012). Successes and challenges in the implementation and application of telemedicine in the eastern province of Saudi Arabia. Perspect Health Inf Manag.

[52] Uscher-Pines L, Kahn JM (2014). Barriers and facilitators to pediatric emergency telemedicine in the United States. Telemed J E Health.

[53] Girault A, Ferrua M, Lalloué B, Sicotte C, Fourcade A, Yatim F, Hébert G, Di Palma M, Minvielle E (2015). Internet-based technologies to improve cancer care coordination: current use and attitudes among cancer patients. Eur J Cancer.

[54] Hofstede J, de Bie J, van Wijngaarden B, Heijmans M (2014). Knowledge, use and attitude toward eHealth among patients with chronic lung diseases. Int J Med Inform.

[55] Edwards L, Thomas C, Gregory A, Yardley L, O'Cathain A, Montgomery AA, Salisbury C (2014). Are people with chronic diseases interested in using telehealth? A cross-sectional postal survey. J Med Internet Res.

[56] Alaboudi A, Atkins A, Sharp B, Balkhair A, Alzahrani M, Sunbul T (2016). Barriers and challenges in adopting Saudi telemedicine network: the perceptions of decision makers of healthcare facilities in Saudi Arabia. J Infect Public Health.

[57] Albarrak AI, Mohammed R, Almarshoud N, Almujalli L, Aljaeed R, Altuwaijiri S, Albohairy T (2021). Assessment of physician's knowledge, perception and willingness of telemedicine in Riyadh region, Saudi Arabia. J Infect Public Health.

[58] Alghamdi SM, Janaudis-Ferreira T, Alhasani R, Ahmed S (2019). Acceptance, adherence and dropout rates of individuals with COPD approached in telehealth interventions: a protocol for systematic review and meta-analysis. BMJ Open.

[59] Ahmed M, Ahmed S, Osman M (2014). Telemedicine and teleradiology in Saudi Arabia. IOSR J Dent Med Sci.

