# Supplementary material for: Culture-Specific Observations in a Saudi Arabian Digital Home Health Care Program: Focus Group Discussions With Patients and Their Caregivers
Source: J Med Internet Res. 2021 Dec 8;23(12):e26002. doi: 10.2196/26002 (PMC8701718; doi:10.2196/26002)
Supplement: Multimedia Appendix 1 [file jmir_v23i12e26002_app1.docx]

**Focus Group Discussion Guide**

**[30 minutes- registration, number tags, welcome, snacks, consent forms]**

**[10 minutes- introduction, ground rules]**

**Discussion Guide**

# To identify cultural observations pertaining to religion, language, family and social life, as well as customs and traditions in during implementation of RAHAH program in Home Health Care Department.

**[Estimated 40 minutes]**

First, I’d like to ask you a few questions about the things you have noticed to be culturally specific when dealing with patients and their caregivers when using RAHAH for Home Health Care.

| **S. No.** | **Question** | **List of Probes** |
| --- | --- | --- |
| 1. | Can you share with us any previous experience (before RAHAH) you have using any remote technology with health? | - *Opening easy question* |
| 2. | During the RAHAH implementation in Home Healthcare, what are some of the religious experiences you faced with patients and/or their caregivers? | - Perhaps they have religious believes that affect how they accept help or ask for it - Maybe they have some believes related to illness and death |
| 3. | You must have noticed that while dealing with the patients and caregivers they use different languages and communication skills, can you describe some of what you have encountered in that regard? | - Perhaps they have certain Verbal and non-verbal communication/ gestures - Use Cultural Metaphors - Perhaps they are Monolingual - Language barriers - Eye contact |
| 4. | Family plays a big role in Saudi people’s health. What is your experience of the role of family during the implementation of RAHAH in HHC? | - Role in decision making? |
| 5. | Can you describe some observations related to Saudi customs and traditions during the RAHAH implementation in Home Healthcare? | - Stigma - Privacy |

# To identify the patients and/or their caregivers’ characteristics that contribute to these cultural observations.

**[Estimated 20 minutes]**

Moving on, I’d like to ask you a few questions about the patients and/or their caregivers’ characteristics that contribute to the cultural observations we just discussed.

| **S. No.** | **Question** | **List of Probes** |
| --- | --- | --- |
| 6. | So, we discussed as a group some of your cultural experiences with the patients and their caregivers. How would you describe the age or gender of the people in those experiences? | - Gender roles - Age |
| 7. | What other characteristics about the patients and (or) caregivers’ background did you notice? | - Family structure - Educational background of the family - Social and racial origins - Financial status - Rural/urban - The type of work experience |

# To identify how culture affects telehealth home health care.

**[Estimated 10 minutes]**

Lastly, I’d like to know your opinion on how culture is affecting the telehealth home health care experience.

| **S. No.** | **Question** | **List of Probes** |
| --- | --- | --- |
| 8. | From your experience in the pilot of RAHAH, what role is culture playing in the implementation of Home Healthcare? | - Is it facilitating - Is it hindering |
| 9. | Overall, from your experience in the pilot of RAHAH, do you think cultural sensitivity is important in the implementation at Home Healthcare? | - Can you suggest some ways to improve cultural sensitivity |

# Concluding Remarks

# [Estimated 5 minutes]
